# Estimating Group Stress Level by Measuring Body Motion

**DOI:** 10.3389/fpsyg.2021.634722

**Published:** 2021-04-01

**Authors:** Satomi Tsuji, Nobuo Sato, Koji Ara, Kazuo Yano

**Affiliations:** ^1^Hitachi, Ltd., Research & Development Group, Tokyo, Japan; ^2^Hitachi, Ltd., Tokyo, Japan

**Keywords:** group stress level, body motion, wearable sensor, duration distribution, work satisfaction, organizational management

## Abstract

Understanding employee stress has become a key issue for top management for corporate growth and risk reduction. So far, annual employee satisfaction surveys (ESs) have been conducted to assess the soundness of an organization. However, since it is difficult to collect questionnaires quantitatively and continuously, there is a need for a practical method that can be used to frequently measure group stress levels with a small burden on employees. We propose such a method and evaluated four combinations of approaches, using activity/rest duration distributions from body motion data and generating estimation models on an individual/group basis. The optimal result was obtained when modeling was made on a group basis by using the activity duration distribution (*r* = 0.928, *p* < 0.001, estimation error: 1.36%), making it possible to assess the degree of the stress of employees quantitatively and easily, and this showed the possibility of this method being useful as a management guide for companies.

## Introduction

In recent years, not only improving efficiency but also reducing employee stress and improving well-being are being recognized as corporate management issues. Because stress interferes with the creativity of employees and increases the turnover rate (Avey et al., 2009), it hinders the growth potential of companies. Employee satisfaction surveys (ESs) are a widely practiced method for grasping the state of an organization. ESs have questions about relationships with supervisors and colleagues at work, motivation, stress, etc. It is common for all employees to answer one once or twice a year. The average value and standard deviation of each department or business unit in the results are calculated and used to decide workplace strategies and personnel policies (Harter et al., 2002). However, the problem with using a questionnaire is that data cannot be collected continuously at short intervals. As a result, decision making may be delayed without noticing an increase in risks. The reason continuous collection is not possible is that the recovery rate and reliability of the answers decrease as the same question is repeated. In addition, methods of measuring human stress by using physiological indicators such as saliva and blood are already known (Booij et al., 2015; Ogino et al., 2017). However, due to the burden and cost of physiological index methods, they are not suitable for collecting long-term data from many employees. For the above reasons, there has been a need for a means of measuring the stress level of an organization with many employees continuously and objectively without interrupting daily work.

The purpose of this study is to estimate the average degree of stress at work by using acceleration data from wearable terminals. Teams or team members wear wearable terminals that include acceleration sensors and that collect data on their body movements. However, if this is an official initiative in a company, the company cannot order that employees be measured for 24 h including during their private time. Therefore, to consider practicality, we have to add the constraint of targeting measurement data obtained only during working hours. The reason we focused on body movement is because we had the following prior research. Although people tend to think that they consciously understand and control themselves, Pentland (2010) proposed the idea of the “honest signal,” which is a non-verbal and unconscious signal made by the body that includes an enormous amount of information about humans themselves. In fact, Nakamura reported that stress differences appear in statistical distributions of body activity (Nakamura et al., 2007). Furthermore, a scaled distribution of mice showed the same tendency as that of humans (Nakamura et al., 2013). These findings support the existence of universal honest signals among animals.

The conventional study (Nakamura et al., 2007) does not describe a method of identifying the degree of stress in healthy people and the case of using data obtained only during working hours. This study extends Nakamura's study (Nakamura et al., 2007), assuming that it will be used in an actual workplace as an alternative to the ESs. If this is realized, the following added value can be expected in management. For example, changes in the average stress level of a department can be monitored daily, and when it increases, managers can quickly notice and intervene. Also, there has been no way to collect continuous stress data in the same organization. However, statistical analysis of continuous data linked to employee work and activity records will likely reveal the factors that affect stress. It is expected that such added value will be welcomed by many companies.

The outline of this paper is as follows. Conventional research and the contribution of this research are described in Section Related Work and Contribution. In Section Method, four approaches of the proposed method are proposed. They are applied to experimental data in Section Experiments, and the evaluation results for the estimation accuracy of each are described. In Section Discussion, we discuss the reason for the approach that obtained the highest estimation accuracy and describe the limitations of this research and future issues. Finally, we conclude in Section Conclusion.

## Related Work and Contribution

### Related Work

Nakamura found a universal law of physical movement (Nakamura et al., 2007). The specific procedure is as follows. The frequency obtained by a wristband-type acceleration sensor is divided into static or active states with a pre-determined threshold. It was also shown that the cumulative proportion distribution of the duration of the static state follows a power law, while that of the duration of the active state obeys stretched exponential functions. Although the scale is different, it has been confirmed that the movement patterns of a mouse (Nakamura et al., 2013) and ant (Hayashi et al., 2015) follow the same distribution, indicating that the law is likely to be common to animals as well as humans. Furthermore, the same study (Nakamura et al., 2007) describes the finding that differences in depressed patients and healthy individuals appeared in the slope of the resting duration distribution and that the distributions of resting duration and activity duration are independent.

Other studies have shown that the flow conditions when people are immersed such as in thinking, desk work, and writing and the excitement of conference participants appear in acceleration data that measures physical movement (Ara et al., 2009; Olguin et al., 2009; Akitomi et al., 2013). In addition, Smarr et al. (2016) indicates that compressing three-axis data into one axis is sufficient for estimating circadian rhythm by using a wristband-type acceleration sensor. This implies that the acceleration of the body contains a large amount of information. Furthermore, as techniques for estimating stress with something other than acceleration, there are techniques using the pressure in rhythm or key strokes during typing (Nozawa et al., 2013) and those using facial expressions, voice, and heartbeats (Jovanov et al., 2003; Pavlidis et al., 2007; Mitsuyoshi, 2015).

In addition, new services using email transmission/reception and chat log analysis, smile detection technology, etc. have been proposed for managers and human resources (Reilly, 2018). This suggests that management has a high need for more frequent understanding of the health of an organization. However, ways have not been sufficiently considered yet of continuously feeding back the status of a workplace without putting a burden on the employees.

### Contribution

The purpose of this study is to estimate the average degree of stress at work by using acceleration data from wearable terminals. The novelty is that doing so estimates the degree of stress in a workable healthy population and has the restriction of using only measurement data obtained during working hours. This contributes to quantifying the health of a workplace more frequently than the ESs.

## Methods

We propose a method that extracts the duration of rest and the duration of activity from an acceleration sensor attached to the body and focuses on the slope of each cumulative distribution fitted to a function.

Based on Pentland's suggestion that unconscious signals reveal various human characteristics, we hypothesize that information that enable us to estimate the degree of stress is hidden in human movement data. Therefore, we adopt the two mathematical pattern of physical movement called power law of static duration and stretched exponential function of active duration that Nakamura have found. Furthermore, since we hypothesized that stress at work is not only individual-dependent but also a collective phenomenon, we adopted two methods, one is to aggregate by individual and the other is to aggregate by organization. In this paper, we evaluate the four approaches that conbination of two mathmatical pattern and two aggregation way as shown in Table 1.

**Table 1:** Four approaches as hypotheses.

**Approach**	**Si**	**Sg**	**Ai**	**Ag**
Data	Static (resting) duration		Active duration	
Fitting function	Power law		Stretched exponential function	
Feature value	Slope (γ)		Slope (β)	
Modeling unit	Individual	Group	Individual	Group

Nakamura et al. (2007) showed that the cumulative distribution of resting duration can be fitted with power law (1), and that of the active duration can be fitted with stretched exponential function (2).

(1) $$\begin{array}{l} P_{c}\left(x\geq T\right)\;=\;{\alpha \cdot T}^{\left(-\gamma \right)}\;\left(\alpha ,\;\gamma \;:\;const.\right) \end{array}$$

(2) $$\begin{array}{l} P_{c\;}\left(x\geq T\right)\;=\exp\left({-\alpha \cdot T}^{\beta }\right)\;\left(\alpha ,\;\beta \;:\;const.\right)\\ \;=\exp{\left(-\alpha^{\frac{1}{\beta }}\cdot T\right)}^{\beta }\\ \;\Leftrightarrow \;\ln\;P_{c}=-{\left(\alpha^{\frac{1}{\beta }}\cdot T\right)}^{\beta }\\ \;=-{\left(\alpha^{\prime }\cdot T\right)}^{\beta }\;\left(\alpha^{\prime }=\alpha^{\frac{1}{\beta }}\right) \end{array}$$

On the basis of this, we will examine and evaluate the four approaches shown in Table 1 as hypotheses. First, two patterns are provided using the distribution of (S) static/resting duration and (A) activity duration; then, two patterns are provided. One (i) obtains an average of a group after creating a stress-estimation model for each individual in the group, and the other (g) creates a stress-estimation model for the entire group as a whole. By combining these, there are four possible approaches: approach Si (static and individual), which creates a model on an individual basis with static data, approach Sg (static and group) with a group model and static data, approach Ai (active and individual) with an individual model and active data, and approach Ag (active and group) with a group model and active data. Those using static duration data adopt γ, which indicates the slope of the power law, as a feature of the model. Those using active duration data adopt β, which indicates the slope of the stretched exponential function.

**Figure 1:**
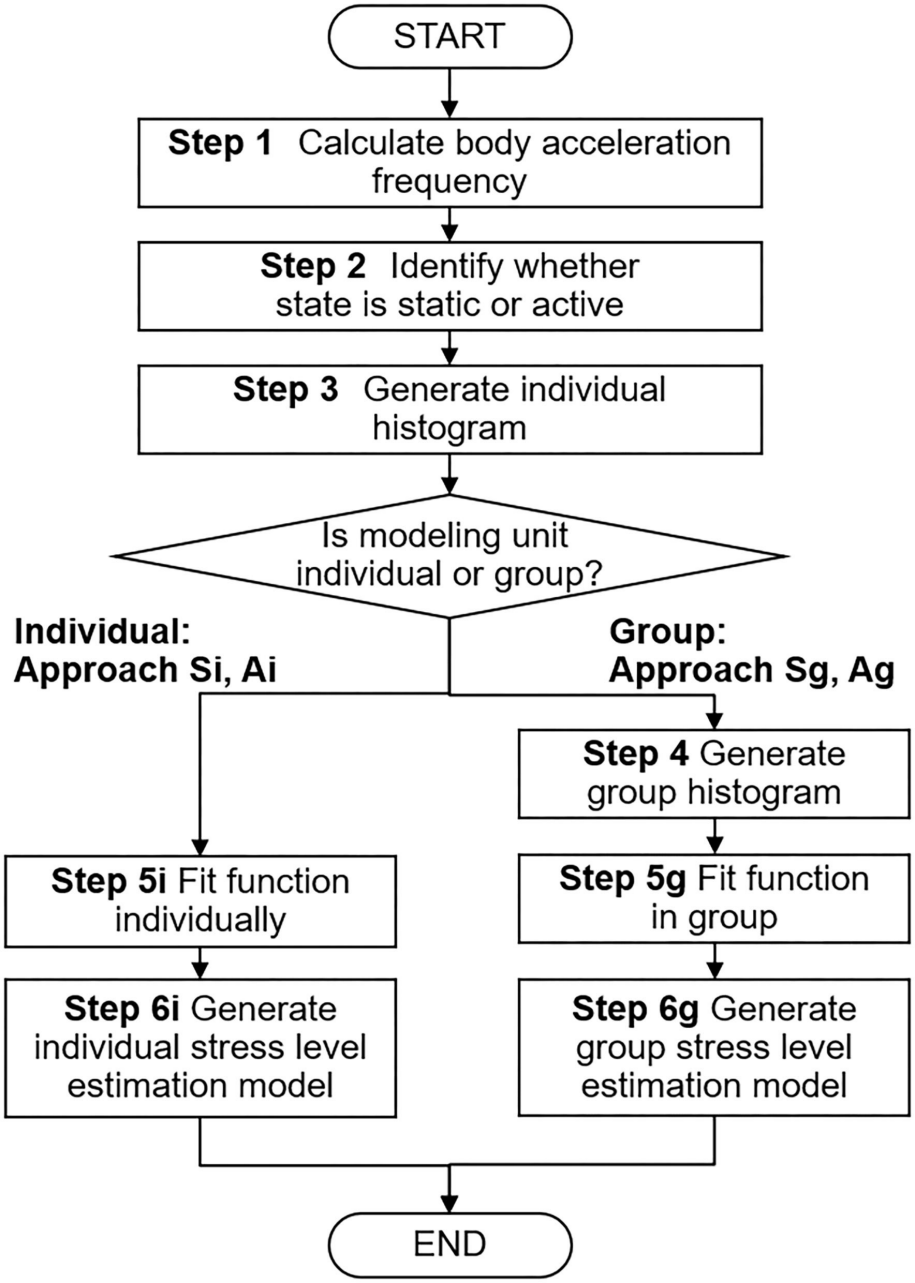
Flowchart of four approaches to estimated model generation. Individual unit models (approach Si or Ai) proceed to Steps 1, 2, 3, 5i, and 6i, and group unit models (approach Sg or Ag) proceed to Steps 1, 2, 3, 4, 5g, and 6g. In Steps 5i and 5g, approaches Si and Sg use power law, and approaches Ai and Ag use stretched exponential function for function fitting.

A flowchart of the four approaches to estimated model generation is shown in Figure 1. The group unit models (approach Sg or Ag) proceed to Steps 1–5g, and 6g, and the individual unit models (approach Si or Ai) proceed to Steps 1–5i, and 6i. The process of each step is described below.

### Step 1: Calculating Body Acceleration Frequency

The frequency per minute is calculated from three-axis acceleration sensor data. After the data are transformed to one-axis data, a high pass filter is applied, and the frequency is obtained by counting the number of zero crosses per minute.

### Step 2: Identifying Whether State Is Active or Resting

A state is identified as “active” or “resting (static)” every 1 min by judging if the acceleration frequency is above or below a pre-defined threshold. Nakamura's study (Nakamura et al., 2007) revealed that the definition of threshold does not affect the function fitting in Steps 5i and 5g because the distributions follow universal laws. Therefore, we defined a common threshold for all subjects.

### Step 3: Generating Histogram of Individual Duration

Histograms are generated by counting the number of occurrences per active duration *T*_*A*_ in the data of each individual *i*. Similarly, histograms of static duration *T*_*S*_ are also generated.

### Step 4: Generating Histogram of Group Duration

This step is performed only in the case of Approaches Sg and Ag. Individual histograms are summed at each T to calculate the cumulative occurrence probability of the population at T to obtain a cumulative distribution function *P*_*c*_.

### Steps 5i, 5g: Fit Function

According to the research by Nakamura et al. (2007), in Step 5i, the cumulative occurrence ratio of the static duration is fitted with power law (1), and in Step 5g, that of the active duration is fitted with stretched exponential function (2). As a result, constants γ and β indicating the slope of each distribution are calculated. The fitting is performed so as to minimize the sum of absolute values of logarithmic differences in the *y*-axis direction.

### Steps 6i, 6g: Generating Model for Estimating Stress Level

In each approach, a simple regression model for estimating the degree of stress is generated. Here, the value of a stress questionnaire used as a reference is an objective variable, and the inclination of each distribution is an explanatory variable. The datasets used for model generation are in individual units for Approaches Si and Ai and in group units for Approaches Sg and Ag. Therefore, in the case of a group unit, the average of the questionnaire values is used. Also, the slope value is γ for Approaches Si and Sg and β for Approaches Ai and Ag.

## Experiments

### Method of Measurement

**Figure 2:**
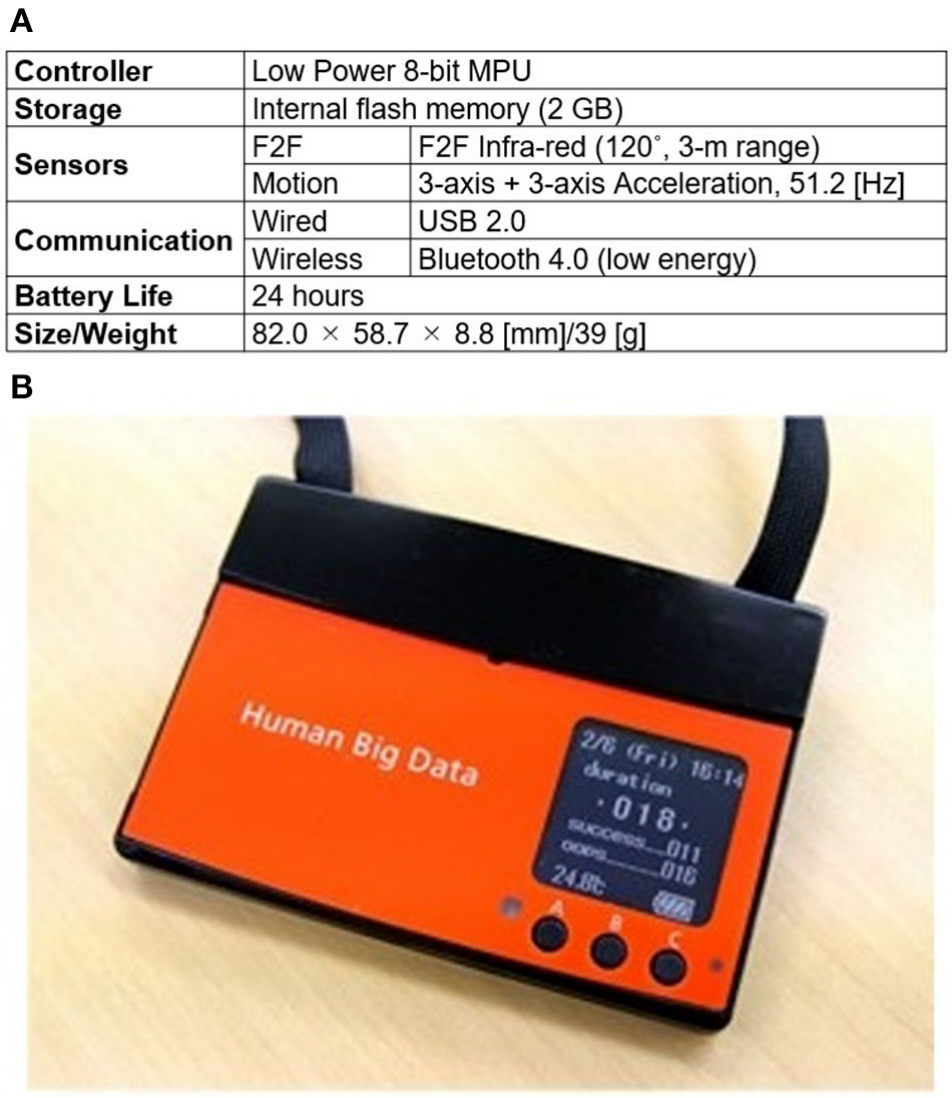
Name-tag shaped wearable sensor node. Nodes continuously measure owner's body motion and face-to-face communication in workplace without interrupting work. Accelerometer in nodes measures body motion at frequency of 51.2 (Hz). Face-to-face communication is detected by transmitting infrared signals between sensor nodes when they face each other at about three meters. **(A)** Specifications **(B)** Appearance This image has been previously published. https://dl.acm.org/doi/fullHtml/10.1145/3358695.3360923.

To measure human behavior in the workplace without interrupting work, we chose a name-tag shaped wearable sensor node (Wakisaka et al., 2009) (Figure 2). Workers put the nodes on when they arrive at work and work as usual with them on while in their office. The nodes are stored in a cradle while the owners are away from the office, where they stop sensing. Thus, the nodes continuously measures an owner's face-to-face communication and body motion in the workplace. Face-to-face communication is detected by transmitting infrared signals between sensor nodes when they face each other at about 3 m. An accelerometer in the nodes measures body motion at a frequency of 51.2 (Hz) and can detect slight movements such as keyboard typing. Moreover, the threshold value that divides static/active in Step 2 of Figure 1 is the minimum frequency that can be detected by this sensor node. In other words, the state is classified as static only when the worker has almost completely stopped.

### Reference of Value

The Center for Epidemiologic Studies Depression (CES-D) questionnaire was adopted as the reference in Steps 6i and 6g (Radloff, 1977; Hann et al., 1999). This is a questionnaire developed by the National Institute of Mental Health for screening depressive conditions and is widely adopted by psychiatrists. The questionnaire has 20 questions such as “I felt depressed,” “My sleep was restless,” and “I was happy (inverted scale).” Respondents look back over the past week and respond in four stages, numbered 0–3, to the number of days they felt that way. As a result, the depression scale of a respondent is calculated by adding together these scores on the questionnaire and creating an integer from 0 to 60. Although the cut-off point for suspected depression on the CES-D is 16, it has been reported that almost 30% of Japanese adults score 16 points or more, which tends to be overestimated compared to the actual prevalence of depression in Japan (Kaneita et al., 2006). Therefore, a cut-off of 26 points has been proposed in Japan. Although the CES-D is not a questionnaire designed to measure the level of stress at work, it is a strong reflection of the level of stress in the workplace because it assesses the subjective perceptions felt as a result of work that takes up about half of the weekday. Therefore, in this paper we consider the value of CES-D to be the stress level of the worker, and furthermore, we consider the average of the group members' CES-D values to be the stress level of the whole group.

### Research Participants

We used data acquired by 10 companies in Japan with 486 people (average of 48.6 ± 29.2 people). Table 2 shows the industry type and job type (department). The target organizations with common characteristics that they are desk work with a little outing were selected. Additionally, several managers and secretaries were included since the experiment participants were designated as whole of departments. Although we could not obtain information on age and gender of the participants, all are in their 20s and 60s. In addition, all employees in the selected organization were informed of the purpose of the experiment and data usage and asked for their consent to participate. Then those who agreed became the participants of this experiment. Only a few people from each organization who disagreed did not wear sensors or answer questionnaires. Since there were few outings, most sensor data taken during working hours were able to be acquired. Sensor data for 1 week including or immediately before the questionnaire response date were used for evaluation of the experiment.

**Table 2:** Research participants.

**Company**	**Industry**	**Department**
A	Finance	Planning
B	Finance	Planning
C	Manufacturing	Engineering
D	Manufacturing	Engineering
E	Manufacturing	Engineering
F	Manufacturing	Engineering
G	Software	Engineering
H	Software	Engineering
I	Software	Engineering
J	Software	Engineering

*Total number of participants was 486 in 10 companies (average of 48.6 ± 29.2 people). Target organizations were selected on condition that work was done inside and at desks*.

### Results

**Figure 3:**
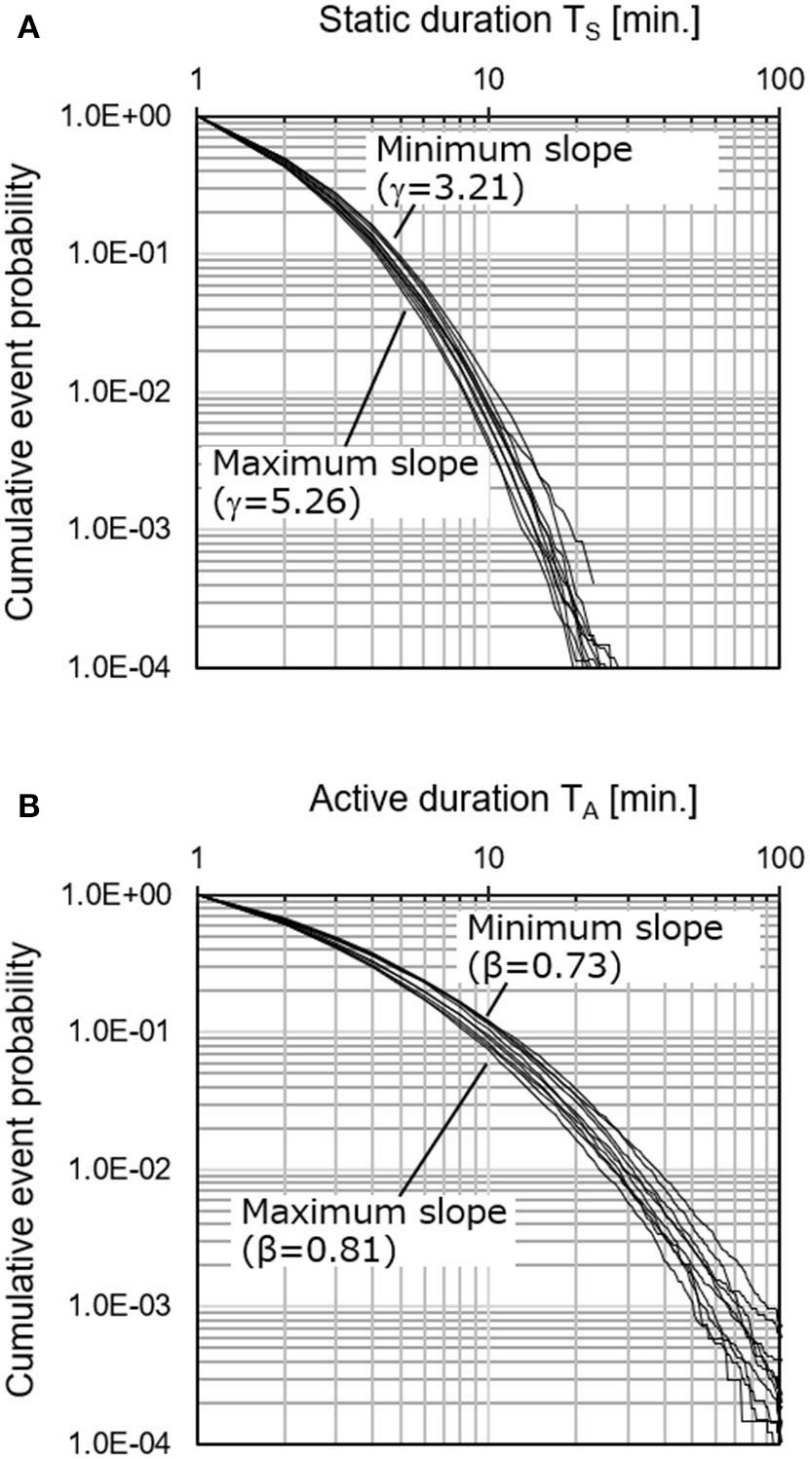
Cumulative occurrence ratio distribution of static and activity duration of 10 companies acquired by name-tag shaped sensor node. Static distribution **(A)** dropped linearly, and difference between 10 groups was small, whereas active distribution **(B)** decreased with gentle curve with differences between groups. **(A)** Static (Resting) Duration **(B)** Active Duration.

Figure 3 shows the cumulative occurrence ratio distribution of the static and activity duration of the 10 companies acquired by the name-tag shaped sensor node. The static distribution in Figure 3A dropped linearly, and the difference between the 10 groups was small, whereas the active distribution in Figure 3B decreased with a gentle curve with the differences between groups.

**Figure 4:**
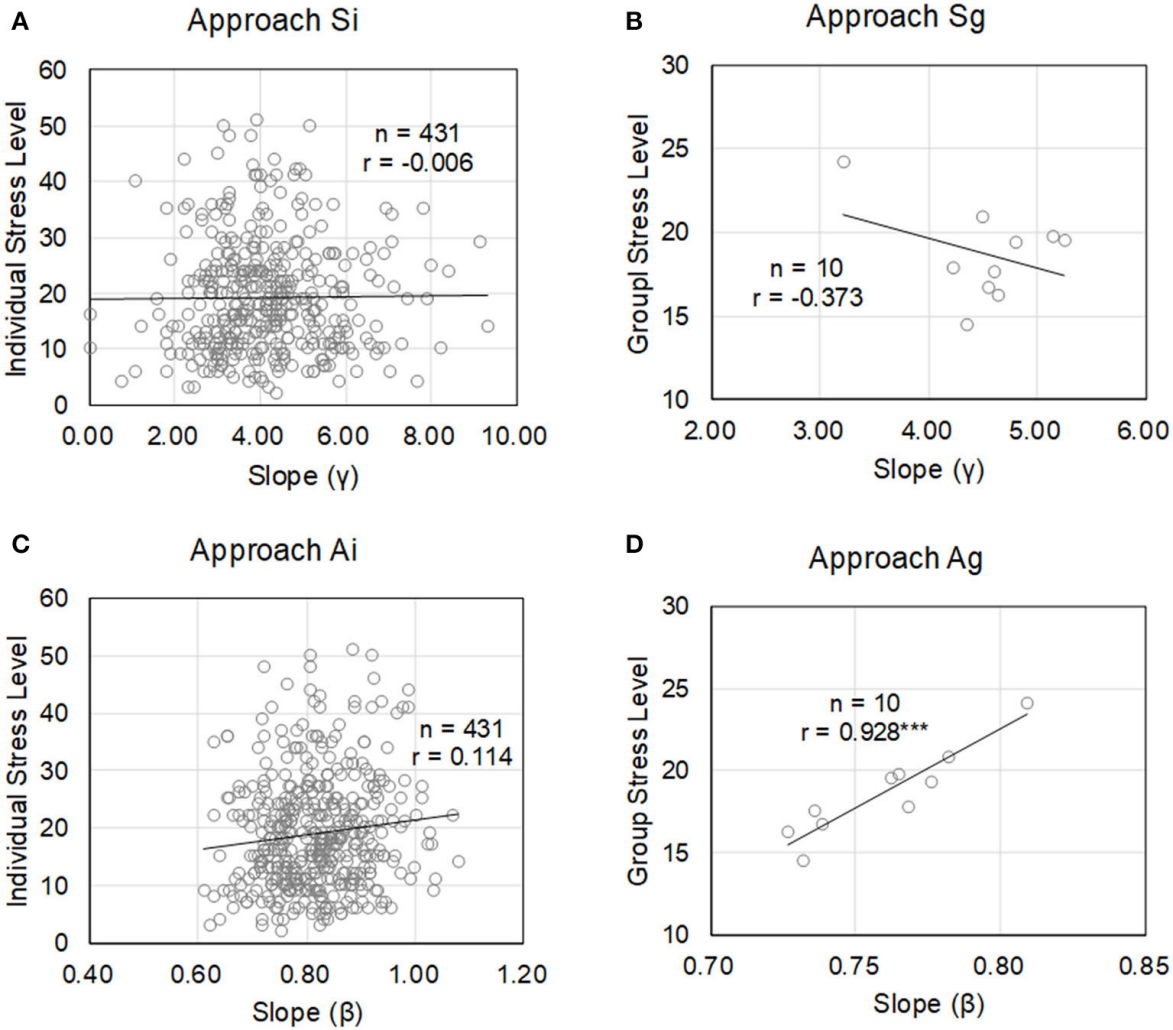
Relationship between slope of each static or active duration distribution and stress level by questionnaire in four approaches. Only Approach Ag showed that the slope can estimate the stress level. **(A)** Approach Si **(B)** Approach Sg **(C)** Approach Ai **(D)** Approach Ag.

The evaluation results for the four approaches are shown in Table 3 and Figure 4. The most accurate approach was Ag, which generates an estimation model on a group basis by using active duration. The resulting correlation coefficient r of this model was 0.928, and the error rate was 1.36%. Since the significance level was *p* < 0.001, the accuracy of this model was sufficiently effective for estimating the average degree of stress of the population. None of the other approaches reached a significance level of *p* < 0.05. As obtained with Equation (3) of Approach Ag, constant *a* had a positive value. That is, this means that the average stress levels tended to be higher as the slopes β in Figure 3B became steeper.

(3) $$\begin{array}{r} Estimated\;group\;stress\;level\;=a\cdot \beta \;+b\;\\ \left(a,\;b\;:\;const.\right) \end{array}$$

**Table 3:** Result of estimating group stress level.

**Approach**	**Si**	**Sg**	**Ai**	**Ag**
Sample size *n*	431	10	431	10
Correlation coefficient *r*	−0.006	−0.373	0.114	0.928^***^
Average error	1.92	2.19	1.85	0.82
Average error rate	3.20%	3.64%	3.08%	1.36%

*** *p < 0.001*.

*Approaches Si, Ai calculated estimated stress level from body rhythm for each individual and calculated average value of group and used it as estimated average stress level of group. Approaches Sg, Ag directly estimated average stress level of group as described in Step 6g. Finally, difference between these estimated average group stresses and those estimated with questionnaire was calculated as error. Correlation coefficient between estimated stress level and that of questionnaire was r. Also, in each approach, cross validation for 10 divisions was performed to evaluate prediction accuracy of estimated models, and average errors of 10 trials were output. Average error rate is value of average error divided by 60, which is difference between maximum and minimum of CES-D questionnaire*.

## Discussion

### What Physical Activity Duration Represents

The average stress level was able to be estimated by approach Ag, which made a model comprised of activity duration distribution and group unit. In this section, we discuss the reasons.

Nakamura's previous study (Nakamura et al., 2007) stated that there was a difference in the slope of the resting duration distribution between depressed patients and healthy people, while there was no difference in the active duration. However, in this study, a difference in terms of the degree of stress did not appear for either Approach Si or Sg using static duration. The reason a difference does not appear in the static duration distribution in our example is considered to be due to the measured hours. In Nakamura's previous study, they performed measurements for 24 h continuously for several days, but in our experiment, only working hours during the daytime were used. In other words, it is thought that the frequency of the occurrence of the resting state during sleep is a strong factor that separates depressed patients from healthy people. As shown in Figure 3A, it is considered that a difference did not appear because a long-lasting static state is less likely to occur compared with the active state in the data of the workplace. Here, although the previous research (wristband type) and this research (name-tag type) are different in terms of the form of the sensor being worn, the movement of the arms and that of the trunk are linked. In addition, since the universal characteristics of the distributions of static and active durations were reproduced, it is considered that the form does not greatly affect the difference in the distribution inclination.

It is an interesting question why the active duration was effective. Comparing the results of Approaches Ai and Ag, Ai, which generated estimation models with an individual unit, was less accurate than Ag with its group unit. From here, it is assumed that Ag is an approach that treats a group as one “closed system.” Hypothetically, we consider that the slope of the active duration distribution represents interaction with others, i.e., the influence others exert on each other or being influenced by other people's stress. This will explain the following. Estimation errors did not occur because the sum of the active durations, which are combinations of each individual's stress plus the stress each individual received from others, of each group member and the sum of the questionnaire results were the same because they were obtained for the same system in Ag. However, in Ai, where the individual is one system, the active duration represents the intra-system effect, while the questionnaire is an internal event of the system; this is why a larger estimation error occurred between body motion and the questionnaire. Additional experiments were performed to confirm this hypothesis.

The subject was Company A in Table 2. Company A consisted of 38 members from three teams, A1, A2, and A3 (13, 14, and 11 members each). We prepared a data set of sensor data and CES-D questionnaire responses for each of three consecutive weeks. In additional experiments, each week, each team was treated as one group, which produced a total of nine group data sets. It was assumed that people should stay close to each other at the same time in order for interactions to occur within the group. Therefore, we shuffled the data of three teams and 3 weeks to generate nine virtual groups and evaluate the estimation accuracy of the average stress degree. The nine groups were randomly divided into three, and the following two patterns of virtual groups were regenerated.

- *Group shuffled:* Three different group pieces were aggregated for the same week, and nine virtual groups were generated.
- *Week shuffled:* One of three different weeks per piece for the same group were aggregated, and nine virtual groups were generated.

Table 4 shows the result of applying approach Ag for both types of virtual data.

**Table 4:** Average error of applying approach Ag for shuffled virtual team.

**Group type**	**Actual**	**(a) Group**	**(b) Week**
	**group**	**shuffled**	**shuffled**
Average error	1.19	3.82	5.28

*Result of applying approach Ag for both types of virtual data. Both virtual groups (a) and (b) had worse accuracy than that of actual group*.

Here, we discuss the inner/inter-group communication of the participants. Table 5 shows the dyad ratio, which was calculated for communication done for more than 15 min per day. It shows that Company A had structural characteristics showing that the people had much inner-group communication but little inter-group communication.

**Table 5:** Communication ratio between teams.

	**Team A1**	**Team A2**	**Team A3**
Team A1	**0.321**	-	-
Team A2	0.104	**0.648**	-
Team A3	0.084	0.104	**0.673**

*Dyad ratio is calculated for communication done for more than 15 min per day. It shows that company A had structural characteristics showing that people had much inner-group communication but little inter-group communication. The bold values means inner group communication*.

In Table 4, for both virtual groups (a) and (b), the accuracy was significantly worse than the result of using actual group division (average error = 1.19). Also, (b) was less accurate than (a). This is considered to be because there was some interaction between shuffled people for (a), but interaction across a time barrier never occurred for (b). Therefore, it was suggested from the result of the additional experiment that the slope of the active duration distribution of the body reflects the interaction of people working in the same space at the same time.

**Figure 5:**
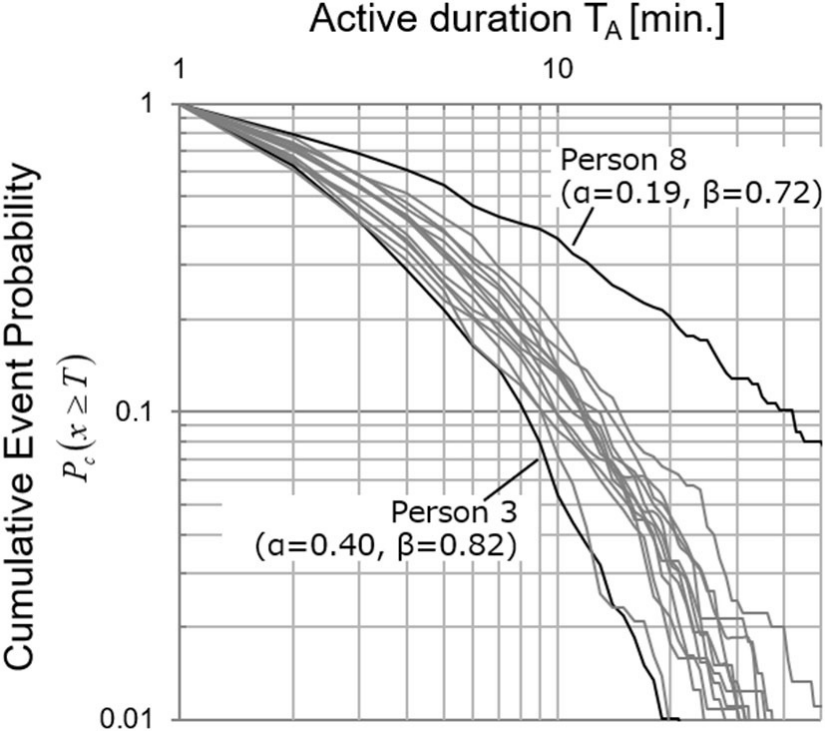
Individual cumulative event probability distributions. Individual cumulative event probability distributions of 14 members of Team A2. This example shows that active state lasting more than 10 min was generated about 10 times as many times as Person 3 for Person 8. This means that physical activity of Person 8 contributed about 10 times that of Person 3 in approach Ag.

Figure 5 shows the distribution of active duration in individual units of all of Team A2, and it can be seen that there were large individual differences. When focusing on time *T* = 10, the *y*-axis value of Person 8 was about 0.4, while that of Person 3 was about 0.04. In other words, it can be said that an active state lasting more than 10 min was generated about 10 times as many times as Person 3 for Person 8. As shown in Step 4 of Figure 1, the cumulative frequency of occurrence for all of the members was the cumulative distribution of Team A2. The physical activity of Person 8 contributed about 10 times that of Person 3 to the distribution of the activity duration of the group. As shown in Step 4 of Figure 1, the sum total of the occurrence frequency of all of the members was the cumulative distribution of Team A2. This means that the physical activity of Person 8 contributed about 10 times that of Person 3 to the distribution of the group. It was already previously confirmed that measures for normalizing and eliminating differences between individuals are not effective in estimating stress in an organization (Tsuji et al., 2017). In addition, the results of Approach Ai show that the difference in the slope β of an individual's active duration distribution was not related to their stress level. In other words, it is thought that there is a meaning in the individual differences in distribution.

So how does this difference in contribution from physical exercise affect the degree of stress in a group? According to the result of Approach Ag in Section Results, it can be said that the average stress is estimated to be lower for organizations with more contributing people, that is, those who tend to keep moving. From this, it was hypothesized that the slope of an individual's activity duration distribution indicates the amount of energy transmitted to the surroundings and that the stress of the person who receives the energy a lot may be reduced. Assuming that a group is a closed system, the total amount of energy transmitted in the group and that of those who receive it should be equal. This hypothesis can explain why the sum of active durations was able to properly estimate the average stress level of a group with the questionnaire in approach Ag. Also, in the other nine companies in Table 2, the communication structures were in line with the definitions of each group, and there was less collaboration across the groups. In other words, they are groups that can be said to be “closed systems,” so it is considered that a high estimation accuracy was obtained in the experiment in Section Experiments. To prove this hypothesis, we think that the propagation path of “energy” and the criteria for judging “closed systems” should be clarified, but these will be issues for the future.

### Significance and Limitations

This study enabled the average stress level of a group to be continuously measured in a practical way. Since the proposed method can be used to perform measurements automatically, what employees have to do is only wear the sensor terminal while working. The advantage compared with ESs used for making conventional management decisions is that the burden on the employees is small. In addition, the frequency of the ES survey is about once a year, but the group stress level can be observed once a week using the results of this study. In other words, a manager can quickly notice and cope with the risks of declining productivity and increasing turnover. An anthropologist, Dunbar, stated that the number of people that could maintain stable social relations was around 150 (Dunbar, 1992). In organizational psychology research, the number of subordinates that a manager can directly manage, the span of control, is broadly known as about 8–12 (Cathcart et al., 2004). However, there is a large number of companies with more than 150 employees and managers with more than 13 subordinates. Internal surveys such as ESs have been conducted to maintain a smooth social relationship in an organization by capturing group conditions that cannot be directly grasped by human executives and managers. In particular, ESs has been used by executives to take over the helm of a company, but fine control cannot be had with annual input. Therefore, in the midst of organizational change, middle managers frequently talk to their subordinates to make up for a lack of information and provide risk understanding and support (Carter et al., 2013). As the speed of organizational change will increase in the future, it is expected that managers will be required to grasp and adjust their subordinates' stress risks more quickly. We believe that this technology can significantly contribute to supporting executives and managers in such situations. Furthermore, by obtaining continuous stress level data, statistical analysis combined with records such as PC logs may also be able to find the cause of workplace stress. In other words, we expect that this study can contribute to discovering not only stress risks but also solutions.

However, the limits of the proposed approach, Ag, are the following three points. First, this study may only guarantee estimation accuracy in a “closed system” group which may be charactaristic of Japanes companies. A closed system refers to an organization, as shown in Table 5, that has more internal communication and less external communication. In the evaluation of Section What Physical Activity Duration Represents, face-to-face communication time was used as an index indicating the amount of interaction between persons. However, it has not been specified whether something that represents the activity duration is transmitted by verbal information in face-to-face communication or non-verbal information such as gaze or voice height. Therefore, it is necessary to evaluate what defines a closed system in the future. For example, it must be considered whether this system can be applied to remote work, shift work sites, and project work sites where human connections change organically. As discussed in Section What Physical Activity Duration Represents, identifying how “energy” that appears in an individual's physical movement affects the stress of others around them will be a clue to solving this problem. The second limitation is that the proposed method guarantees estimation accuracy only for desk work-oriented jobs. Since the proposed method is calculated on the basis of physical movement, there is a possibility that the slope β of the active duration distribution will change for types of work in which there is continual movement while working, such as nursing, retail, and warehouse work. It is necessary to extend the experimental target, which, in this paper, was office workers, and evaluate the robustness of the proposed method to see whether the same method can be applied with the same parameters to these other types of work. The third limitation is the proposed approach is to try to explain stress only with motion sensor in spite of stress at work can be caused by a lot of variables.

## Conclusion

In this paper, we proposed a method for estimating the stress level of a group with a focus on body movement, and we evaluate four approaches. As a result, we confirmed that we could perform estimation with high accuracy by using an approach of generating an estimation model of a group unit by using the active duration distribution (*r* = 0.928, *p* < 0.001, estimation error: 1.36%). The feature of this approach is that it is practical. Since a group stress level can be automatically measured simply by wearing a sensor terminal while working, the burden on the employee is small, even if the level is measured more frequently than the ESs), which is conventionally used for management decisions. The results of this study will enable us to observe the state of an organization about once a week, so managers can quickly notice and cope with the risks of declining productivity and increasing turnover rates. From the above, we confirmed the possibility that this study can contribute to supporting executives and managers in their decision making.

### Future Work

Future work involves the following two points.

- Identifying a mechanism that represents the duration of physical activity and its effect on the stress level of people around a person.
- Robustly evaluating the proposed method in an experiment with groups from other job types.

## Data Availability Statement

The raw data supporting the conclusions of this article will be made available by the authors, without undue reservation.

## Ethics Statement

The studies involving human participants were reviewed and approved by Hitachi, Ltd., Research & Development Group. The patients/participants provided their written informed consent to participate in this study.

## Author Contributions

ST designed the study, analyzed the data, and wrote the manuscript. KA was involved in conceptualizing the study including data collection. KY designed the study and provided feedback. NS was involved in data collection and provided feedback. All authors contributed to the article and approved the submitted version.

## Conflict of Interest

ST, NS, KA, and KY are employed by the company Hitachi, Ltd.
